# The enhancement of tolerance to salt and cold stresses by modifying the redox state and salicylic acid content via the *cytosolic malate dehydrogenase* gene in transgenic apple plants

**DOI:** 10.1111/pbi.12556

**Published:** 2016-03-29

**Authors:** Qing‐Jie Wang, Hong Sun, Qing‐Long Dong, Tian‐Yu Sun, Zhong‐Xin Jin, Yu‐Jin Hao, Yu‐Xin Yao

**Affiliations:** ^1^ State Key Laboratory of Crop BiologyCollege of Horticulture Science and EngineeringShandong Agricultural UniversityTai‐AnShandongChina

**Keywords:** apple (*Malus domestica* B.), *cytosolic malate dehydrogenase* gene, overexpression, salt and cold tolerance, redox state, salicylic acid

## Abstract

In this study, we characterized the role of an apple *cytosolic malate dehydrogenase* gene (*MdcyMDH*) in the tolerance to salt and cold stresses and investigated its regulation mechanism in stress tolerance. The *MdcyMDH* transcript was induced by mild cold and salt treatments, and *MdcyMDH‐*overexpressing apple plants possessed improved cold and salt tolerance compared to wild‐type (WT) plants. A digital gene expression tag profiling analysis revealed that *MdcyMDH* overexpression largely altered some biological processes, including hormone signal transduction, photosynthesis, citrate cycle and oxidation–reduction. Further experiments verified that *MdcyMDH* overexpression modified the mitochondrial and chloroplast metabolisms and elevated the level of reducing power, primarily caused by increased ascorbate and glutathione, as well as the increased ratios of ascorbate/dehydroascorbate and glutathione/glutathione disulphide, under normal and especially stress conditions. Concurrently, the transgenic plants produced a high H_2_O_2_ content, but a low $O_{2}^{\cdot -}$ production rate was observed compared to the WT plants. On the other hand, the transgenic plants accumulated more free and total salicylic acid (SA) than the WT plants under normal and stress conditions. Taken together, *MdcyMDH* conferred the transgenic apple plants a higher stress tolerance by producing more reductive redox states and increasing the SA level; *MdcyMDH* could serve as a target gene to genetically engineer salt‐ and cold‐tolerant trees.

## Introduction

Plant tissues possess multiple isoforms of malate dehydrogenase (MDH, EC 1.1.1.37), which belongs to the group of oxidoreductases and catalyses the interconversion of malate and oxaloacetate (OAA) coupled to the reduction or oxidation of the NAD(H) or NADP(H) pool. NAD‐dependent MDHs are located in the cytosol and in organelles, including plastids, mitochondria, peroxisomes and microbodies; additionally, chloroplasts contain an NADP‐dependent MDH (Gietl, 1992; Scheibe, 2004). In the *Arabidopsis* genome, eight putative NAD‐MDH isoforms have been identified, where two are mitochondrial MDH (mMDH), two are peroxisomal MDH, one is a plastidial MDH, and three have no detectable target sequences and are thought to be cytosolic MDH (cyMDH) (Beeler *et al*., 2014).

The redox state of the cells is especially critical during exposure to abiotic stresses, which are known to induce oxidative stress at the cellular level; under these conditions, regulation of multiple redox and reactive oxygen species (ROS) signals in plants requires a high degree of coordination and balance between the signalling and metabolic pathways in different cellular compartments (Suzuki *et al*., 2012). In green tissues, an important aspect is the chloroplast/cytosol/mitochondrion cooperation under stress to modulate cell redox homeostasis (Raghavendra and Padmasree, 2003). MDH reaction is involved in central metabolism and redox homeostasis between organelle compartments (Tomaz *et al*., 2010). MDH isozymes in the chloroplast inner envelope membrane, stroma, mitochondrial membrane and cytosol are the components of malate/OAA and malate/aspartate (Asp) shuttles (malate valves) (Gietl, 1992; Taniguchi and Miyake, 2012). When plants are subjected to abiotic stresses, changes in the activities of the enzymes of the malate valves and expression levels of the MDH isoforms can be observed (Scheibe, 2004), and malate valves play major roles in reductant export under stress conditions (Taniguchi and Miyake, 2012). Chloroplast NADP‐MDH makes the link between the redox states of the chloroplast and the cytosol and other cell compartments, such as peroxisomes (Heyno *et al*., 2014). In *Arabidopsis*, malate/OAA transporter knockout impairs the malate valve function of chloroplasts, and the knockout plants show enhanced photoinhibition and ROS accumulation in response to a high‐intensity light, due to a greater accumulation of reducing equivalents in the stroma (Lemaire *et al*., 1996). Regarding mMDH, its repression modifies the ascorbate‐mediated link between the energy‐generating processes of respiration and photosynthesis (Nunes‐Nesi *et al*., 2005). Additionally, mMDH lowers leaf respiration and alters photorespiration and plant growth in *Arabidopsis* (Tomaz *et al*., 2010). In contrast, cyMDH is thought to function as a key player in the transfer of reducing equivalents from the chloroplast or mitochondria to other destinations in plant cells (Gietl, 1992; Hara *et al*., 2006; Scheibe, 2004).

On the other hand, plant hormones play central roles in the ability of plants to adapt to abiotic stresses by mediating a wide range of adaptive responses (Peleg and Blumwald, 2011). For example, evidence of the role of salicylic acid (SA) in the oxidative damage generated by NaCl and osmotic stress was reported, and the NPR1‐dependent SA signalling pathway was found to play pivotal roles in enhancing salt and oxidative stress tolerance in *Arabidopsis* (Borsani *et al*., 2001; Jayakannan *et al*., 2015); in contrast, ABA‐dependent and ABA‐independent signalling pathways in response to osmotic stress were widely investigated (Yoshida *et al*., 2014). Additionally, interactions between hormone and redox signalling pathways can control plant growth and cross‐tolerance to stress (Bartoli *et al*., 2013). Up to now, evidence that cyMDH and other MDHs can enhance plant stress tolerance by regulating redox and hormone levels, as well as from their interactions, is still lacking.

Because most of the arable lands are used for growing grain crops, fruit trees are increasingly grown on marginal land. Because fruit trees cannot be rotated to avoid stresses, in contrast to annual crops, they often encounter multiple environmental stresses simultaneously. It has become an emerging challenge for scientists to clarify how fruit trees respond to and grow against adverse environments and to improve the stress tolerance of fruit trees by genetic modification. Presently, it has been reported that *cyMDH* confers superior manganese tolerance by mediating malate synthesis and secretion and inhibiting ROS generation in *Stylosanthes guianensis* (Chen *et al*., 2015). Our previous study also showed that *MdcyMDH* conferred transgenic tomato plants high tolerance to cold and salt stresses alongside with decreased ROS levels (Yao *et al*., 2011a). However, more research is needed to unravel the mechanism of *MdcyMDH* in regulating abiotic stresses. Therefore, this study is aimed to assess the potential application of *MdcyMDH* in engineering salt‐ and cold‐tolerant apple trees and to better understand the mechanism of *MdcyMDH*‐mediated abiotic stress tolerance by modifying redox homeostasis and hormone levels.

## Results

### The identification of *cyMDH* genes and their expression in different tissues and under cold and salt treatments in apple

**Figure 1:**
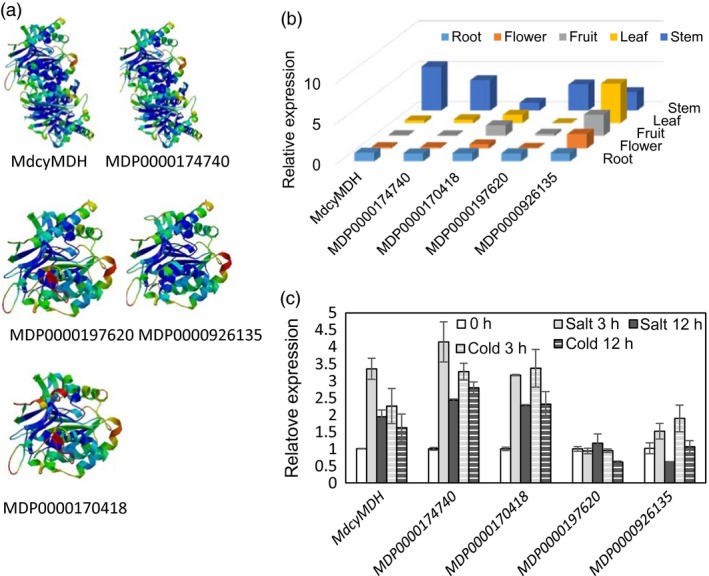
Predicted protein tertiary structures of the different cyMDH isoforms (a) and expression patterns of the *cyMDH* genes in different tissues (b) and in response to 8 °C temperature and 50‐mm salt in the leaves of apple *in vitro* shoot cultures (c). Protein tertiary structures were predicted using the SWISS‐MODEL (http://swissmodel.expasy.org/). Values represent the means ± SD of three replicates.

In our previous study (Yao *et al*., 2008), the *MdcyMDH* (DQ221207) ORF was shown to encode a 332‐aa polypeptide which shared over 90% similarity with cyMDHs from other species, but we failed to locate this gene in the present apple genome database. In contrast, the other four *cyMDH* isoforms were identified from the entire apple genome, and their deduced proteins possessed the highly conserved domains of cytosolic malate dehydrogenase (Figure S1). Highly similar protein tertiary structures were observed between MdcyMDH and MDP0000174740, as well as between MDP0000197620 and MDP0000926135 (Figure 1a). Transcripts of the five *cyMDH* genes could be detected in all tissues, but they exhibited varying expression levels in different tissues; *MdcyMDH*,* MDP0000174740* and *MDP0000197620* were expressed primarily in the stems and roots (Figure 1b,c). *MdcyMDH*,* MDP0000174740* and *MDP0000170418* expression were induced by 50 mm NaCl and temperature of 8 °C (Figure 1c). Therefore, MdcyMDH and MDP0000174740 most likely possessed similar functions in the light of their highly similar tertiary structures, spatial expressions and expression responses to salt and cold in apple.

### Improved tolerance to salt and cold in transgenic apple plants via *MdcyMDH* overexpression

**Figure 2:**
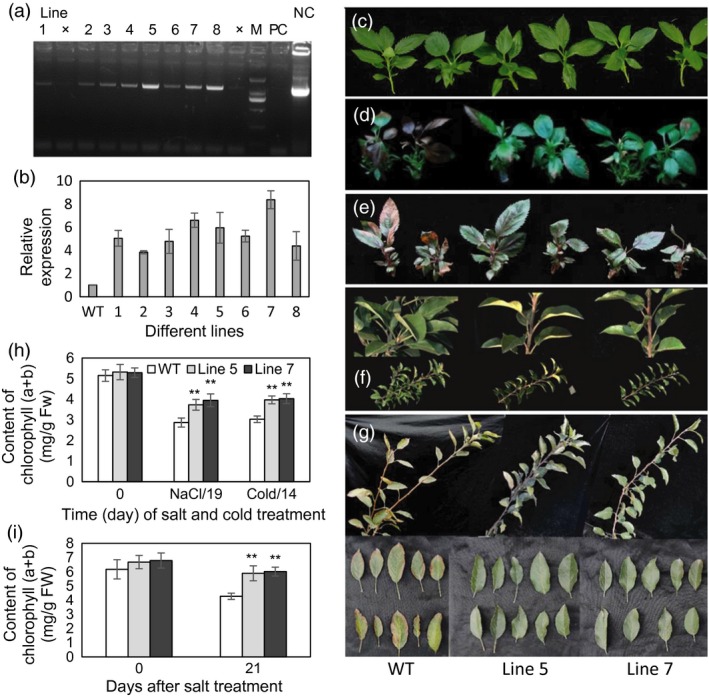
PCR identification of *MdcyMDH*‐overexpressing apple plants and the evaluation of salt and cold tolerance of transgenic plants. (a) PCR identification using DNA templates and the specific primers from *35S* and *MdcyMDH*. (b) *MdcyMDH* expression in the leaves of wild‐type (WT) and transgenic lines. (c–e) WT and transgenic apple *in vitro* shoot cultures at 20 days after a subculture were taken as the control (c); the cultures with the same subculture conditions as the control were subjected to stress treatments, that is at 50‐mm NaCl for 10 days and subsequent 100‐mm NaCl for 9 days (d); at 8 °C for 7 days and subsequent 0 °C for 7 days in a 14‐h light photoperiod (e). (f, g) Shoots and leaves of the 3‐year‐old pot‐cultured WT and transgenic apple trees prior to stress treatments (f) and after 100‐mm NaCl for 14 days and subsequent 150‐mm NaCl for 7 days (g). (h, i) Contents of chlorophyll (a + b) of the leaves from the transgenic apple cultures (h) and trees (i) under normal and stress conditions. Values represent the means ± SD of three replicates. M, DNA marker; NC, negative control (H_2_O); PC, positive control (plasmid DNA of *35S::MdcyMDH*). **Highly significant difference, *P *<* *0.01.

To further characterize the biological function of the *MdcyMDH* gene, the *MdcyMDH*‐overexpressing lines were obtained by agrobacterium‐mediated transformation and confirmed by PCR detection of the target gene (Figure 2a,b). Lines 5 and 7, with high *MdcyMDH* expression level compared to the wild‐type (WT) plants, were selected to be further assayed (Figure 2b). When subjected to 50 mm salt and 8 °C stresses, no obvious phenotype differences were observed between the transgenic and WT apple *in vitro* shoot cultures at 10 days after the treatments; after nine more days of 100 mm NaCl and seven more days of 0 °C treatment, both the transgenic and WT apple cultures showed the necrosis phenotypes, but the transgenic plants exhibited less severe phenotypes (Figure 2d,e). In addition, after 3 weeks of NaCl treatment, the leaves of the 3‐year‐old WT plants exhibited symptom of etiolation and marginal necrosis but the leaves of the transgenic plants exhibited near‐to‐normal colour (Figure 2g). Moreover, the leaves of the transgenic apple cultures and trees possessed a much higher chlorophyll content than the leaves of the WT plants after the stress treatments although the chlorophyll contents were reduced in the WT and transgenic plants when subjected to the stress treatments (Figure 2h,i). Therefore, *MdcyMDH* overexpression enhanced the tolerance of transgenic apple plants to salt and cold stresses.

### Comparison of expression profiles between the WT and *MdcyMDH*‐overexpressing plants

**Figure 3:**
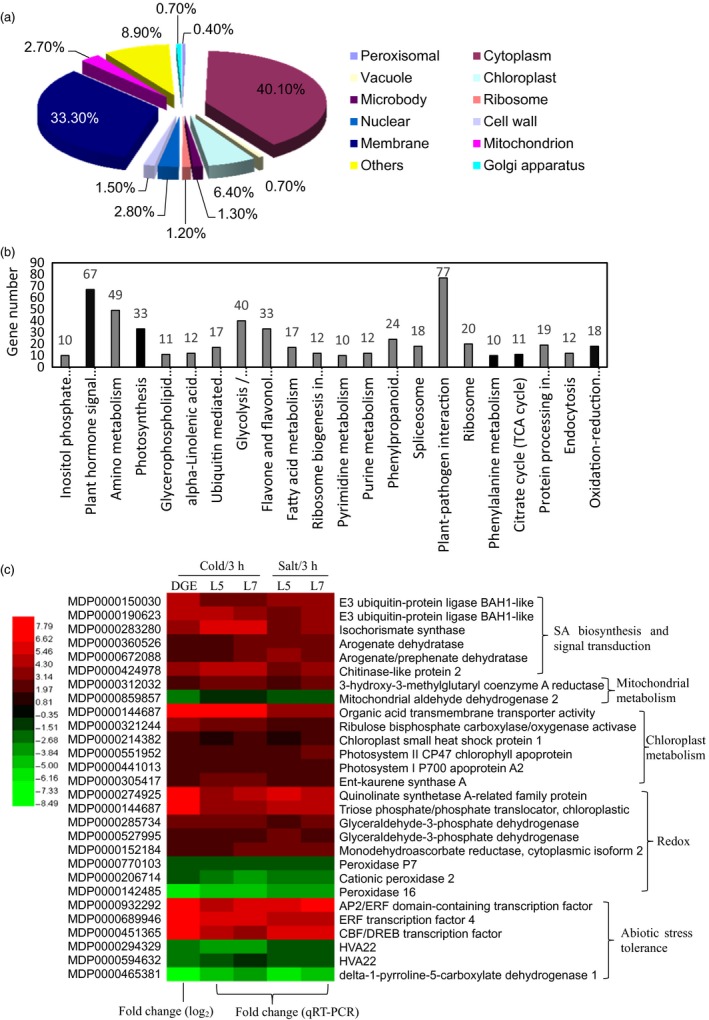
Cell component (a) and biological process (b) of the digital gene expression (DGE) genes classified by Gene Ontology and the most differentially expressed genes related to SA biosynthesis and signalling, mitochondrial and chloroplast metabolism, and redox and abiotic stress tolerance (c). In the heat map of panel (c), the ID and annotation of each gene are provided. Fold changes from qRT‐PCR at 3 h after cold and salt treatments were used to validate the DGE genes and to determine the expression changes of the DGE genes induced by *MdcyMDH* overexpression under stress treatments. Fold change from qRT‐PCR is calculated by comparing the relative expression values of the selected genes in the transgenic and wild‐type (WT) plants. Data are presented as the means of three replicates.

To explore the regulation mechanism of *MdcyMDH* in abiotic stresses, a digital gene expression (DGE) tag profiling analysis between the WT and mixed transgenic plants at 3 h after the cold treatment was conducted to quantify gene changes. It was found that 961 and 958 genes were up‐ and down‐regulated by at least onefold in the transgenic lines, respectively, demonstrating that a massive transcriptional reprogramming took place in the *MdcyMDH*‐overexpressing plants. According to the putative homology to sequences present in public databases, the differentially expressed genes were classified into 12 different cellular component categories; the cytosol contained the most differentially expressed genes (40.1%), followed by the membrane (33.3%), chloroplast (6.4%), nucleus (2.8%) and mitochondria (2.7%) (Figure 3a). All of the DGE genes are associated with 22 biological processes. The two processes of plant–pathogen interaction and plant hormone signal transduction contain the most DGE genes; additionally, photosynthesis, citrate cycle (TCA cycle) and the oxidation–reduction process were also the clearly changed biological processes (Figure 3b). Moreover, the expression levels of the 28 most differentially expressed annotated genes in the cold‐treated shoot cultures were detected by qRT‐PCR, which contained genes related to SA biosynthesis and signal transduction, chloroplast and mitochondrial metabolism, redox process and abiotic stresses; similar expression changes to DGE tag profiles were found, validating the reliability of the DGE genes (Figure 3c). Additionally, the 28 genes exhibited similar expression changes under salt treatment as that under cold treatment (Figure 3c). Besides, most of the above genes were induced by cold and/or salt in the WT and transgenic plants (Figure S2). Therefore, it is suggested that *MdcyMDH* improved the stress tolerance of the transgenic apple plants at least partly via modifying hormone signal transduction, photosynthesis, TCA cycle and the oxidation–reduction process.

### *MdcyMDH* overexpression modulated mitochondrial and chloroplast metabolisms

**Figure 4:**
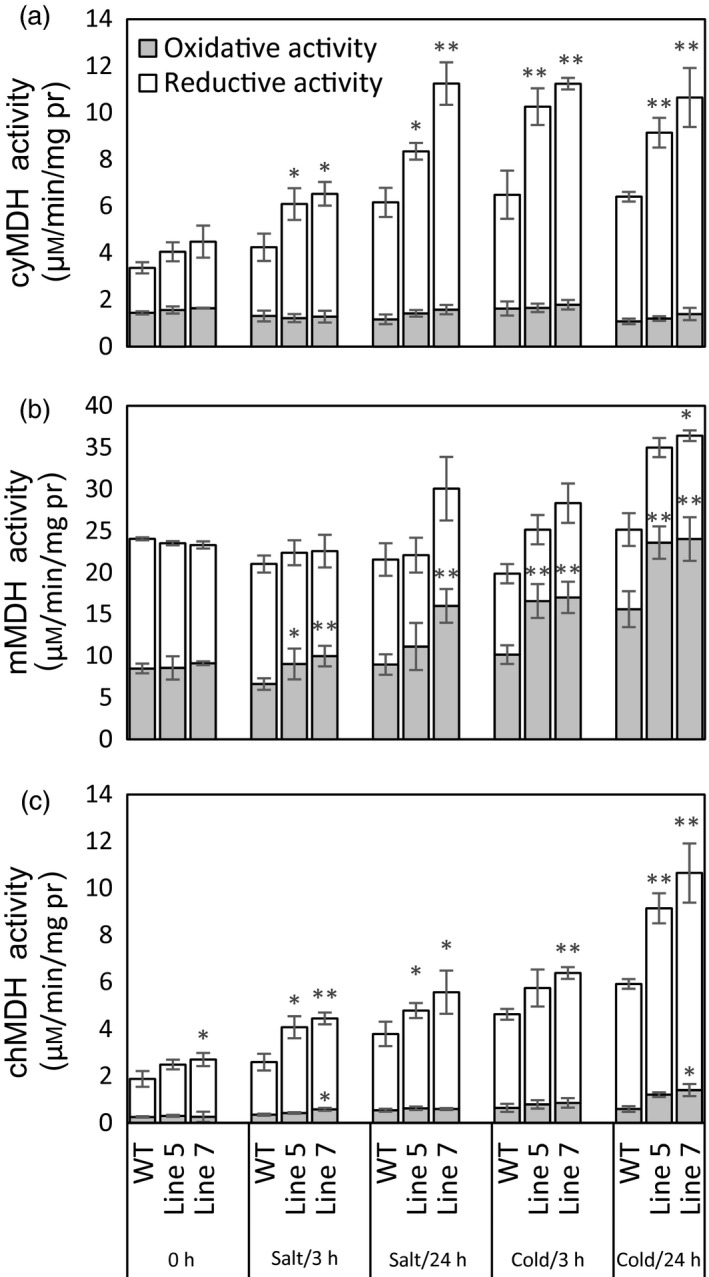
Modifications of reductive and oxidative activities of malate dehydrogenase (MDH) in the cytosol (a), mitochondria (b) and chloroplast (c) in the wild‐type (WT) and transgenic plants in response 50 mm salt and 8 °C stresses. Data are presented as means ± SD (*n* = 3). The difference level was compared between the transgenic lines and WT at the same treatment time. *Significant difference, *P *<* *0.05; **highly significant difference, *P *<* *0.01.

**Figure 5:**
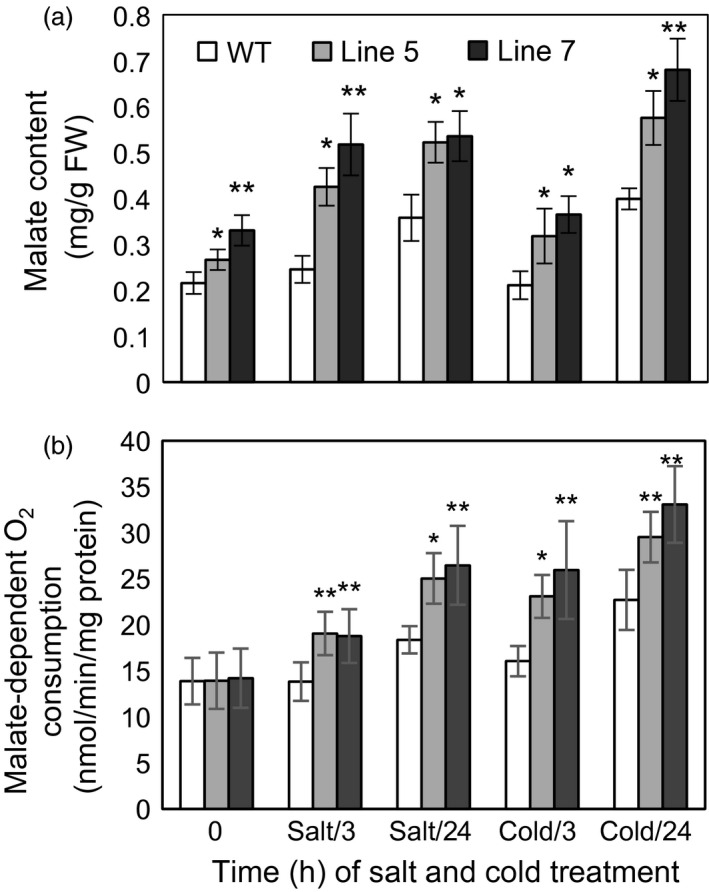
Malate content and malate‐dependent O_2_ consumption in the wild‐type (WT) and transgenic plants under cold and salt treatments. Data are presented as means ± SD (*n* = 3). The difference level was compared between transgenic lines and WT at the same treatment time. *Significant difference, *P *<* *0.05; **highly significant difference, *P *<* *0.01.

First, MDH activities in the cytosol, mitochondria and chloroplast were detected in the *MdcyMDH*‐overexpressing apple *in vitro* shoot cultures (Figure 4a–c). The *MdcyMDH* overexpression increased the reductive activities of cyMDH and chMDH and the oxidative activity of mMDH compared to the WT plants; in contrast, the difference of activities was enlarged and reached a significant level under cold and salt stresses. In contrast, the oxidative activities of cyMDH and chMDH and the reductive activity of mMDH were generally not significantly changed in the transgenic plants compared to those in the WT plants. Moreover, the reductive activities of cyMDH and chMDH and the oxidative activity of mMDH were clearly induced by cold and salt treatment in the WT and transgenic plants (Figure 4a–c). Therefore, it can be suggested that the increases in the cyMDH and chMDH reductive activities and the mMDH oxidative activity contributed favourably to the stress tolerance of apple plants in the transgenic plants. Moreover, the significantly increased malate content indicated that the reductive activity of MDH was strengthened against its oxidative activity in the transgenic plants under normal and stress conditions (Figure 5a).

**Figure 6:**
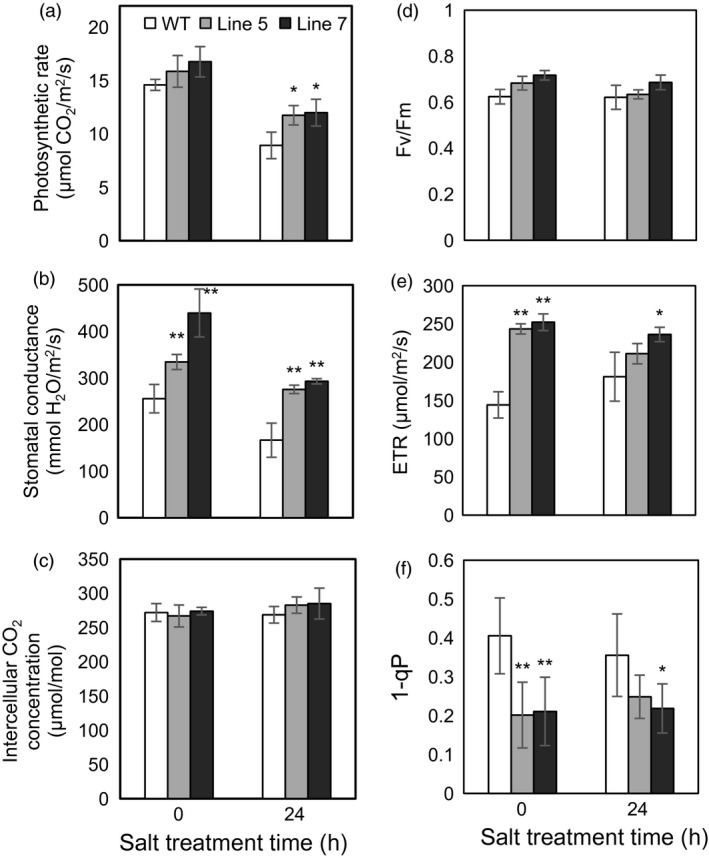
Changes of the photosynthetic rate (a), stomatal conductance (b), intercellular CO_2_ concentration (c), maximum quantum yield of PSII (*F*_v_/*F*_m_) (d), photosynthetic electron transport rate (ETR) (e), and PSII excitation pressure (1−qP) (f) in the leaves of the wild‐type (WT) and transgenic plants at 0 and 24 h after 50‐mm NaCl treatment. Data are presented as means ± SD (*n* = 3). *Significant difference, *P *<* *0.05; **highly significant difference, *P *<* *0.01.

Because the MDH activities in the cytosol, mitochondria and chloroplast were altered in the transgenic plants and MDH is one of the components of the malate/OAA shuttle between the cytosol and other organelles, it was speculated that the mitochondrial and chloroplast metabolisms would be changed. To verify this speculation, mitochondrial and chloroplast metabolisms were detected in the WT and transgenic plants under normal and stress conditions. On the one hand, malate‐dependent oxygen consumption by isolated mitochondria was measured to assess the change in the mitochondrial activity. The results showed that the oxidation capacity of malate was significantly increased in the transgenic plants under cold and salt treatments (Figure 5b), which was consistent with the increased oxidative activity of mMDH (Figure 4b). On the other hand, parameters related to photosynthesis and chlorophyll fluorescence were determined to assess the changes in chloroplast metabolism in the transgenic plants. The results showed that the NaCl treatment induced a decrease in the photosynthetic rate in the WT and transgenic plants; nevertheless, the *MdcyMDH* overexpression produced an increase in the photosynthetic rate and a concomitant increase in the stomatal conductance (Figure 6a,b), suggesting the contribution of stomatal factors to the increased photosynthetic rate in the transgenic plants. However, similar intercellular CO_2_ concentration in the leaves of the WT and transgenic plants also indicated altered activities of the mesophyll by the *MdcyMDH* overexpression (Figure 6c). Compared to the WT plants, *MdcyMDH* overexpression did not significantly influence the photoinhibition of PSII in the light of similar ratios of *F*_v_/*F*_m_ under normal and salt treatment (Figure 6d; Björkman and Demmig, 1987); in contrast, *MdcyMDH* overexpression significantly increased the electron transport rate (ETR), which was used as a quantitative indicator of the electron transport beyond PSII (Maxwell and Johnson, 2000) (Figure 6e). Additionally, the transgenic plants significantly modified the redox level and excitation pressure of PSII, indicated by the altered values of 1−qP (Figure 6f; Maxwell and Johnson, 2000). Taken together, the mitochondrial and chloroplast metabolisms were modified in the *MdcyMDH*‐overexpressing plants.

### Overexpression of *MdcyMDH* increases the reducing power and modifies the ROS level under cold and salt stresses

**Figure 7:**
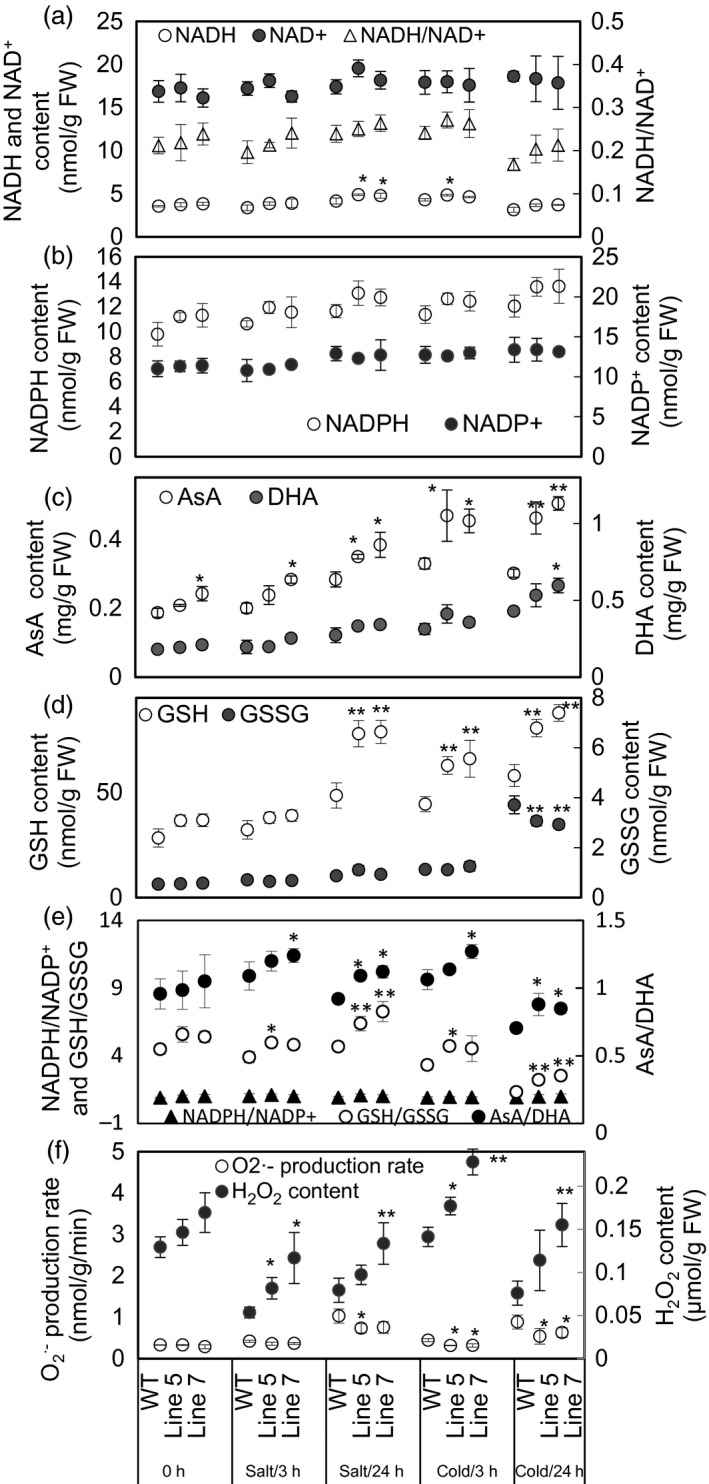
Assays of redox couples and reactive oxygen species (ROS) in the wild‐type (WT) and transgenic plants under 50 mm NaCl and 8 °C treatments. (a) The contents of the reduced form of NADH and the oxidized form of NAD ^+^, as well as the ratio of NADH/NAD ^+^. (b–d) The contents of the reduced form of redox couples (NADPH, AsA and GSH) and the oxidized form of redox couples (NADP ^+^, DHA and GSSG). (e) The ratios of the reduced and oxidative forms of redox couples. (f) The contents of $O_{2}^{\cdot -}$ and H_2_O_2_. Data are presented as means ± SD (*n* = 3). *Significant difference, *P *<* *0.05; **highly significant difference, *P *<* *0.01. The analysis of the significant difference was performed between the transgenic lines and WT plants at the same treatment point.

The three most abundant antioxidants in plant cells, that is NAD(P)H, ascorbate and glutathione (Queval and Noctor, 2007), and their reductive and oxidized forms were determined in the WT and transgenic plants under normal and stress conditions (Figure 7). *MdcyMDH* overexpression increased the NADH levels and the ratios of NADH/NAD^+^ although generally not in a statistically significant manner under normal and stress conditions (Figure 7a). In contrast, the NADPH content was slightly enhanced but the ratios of NADPH/NADP^+^ were almost unchanged (Figure 7b,e). Moreover, accumulation of both reduced ascorbate (AsA) and oxidized ascorbate (DHA) was induced by cold and salt stresses (Figure 7c). The contents of both AsA and DHA increased in the transgenic plants compared to the WT plants; additionally, the increments in AsA and AsA/DHA reached statistical significance at most of the treatment points (Figure 7c,e). Similarly, salt and cold induced the accumulation of oxidized glutathione (GSSG) and especially reduced glutathione (GSH); *MdcyMDH* overexpression significantly promoted the GSH content and GSH/GSSG under stress conditions (Figure 7d,e). Taken together, *MdcyMDH* overexpression increased the reducing power predominantly via enhancing the levels of AsA and GSH, as well as the ratios of AsA/DHA and GSH/GSSG.

On the other hand, the transgenic lines generated a lower $O_{2}^{\cdot -}$ production rate but a higher H_2_O_2_ content than the WT plants under normal and stress conditions (Figure 7f).

### The level of SA is elevated in the transgenic plants

**Figure 8:**
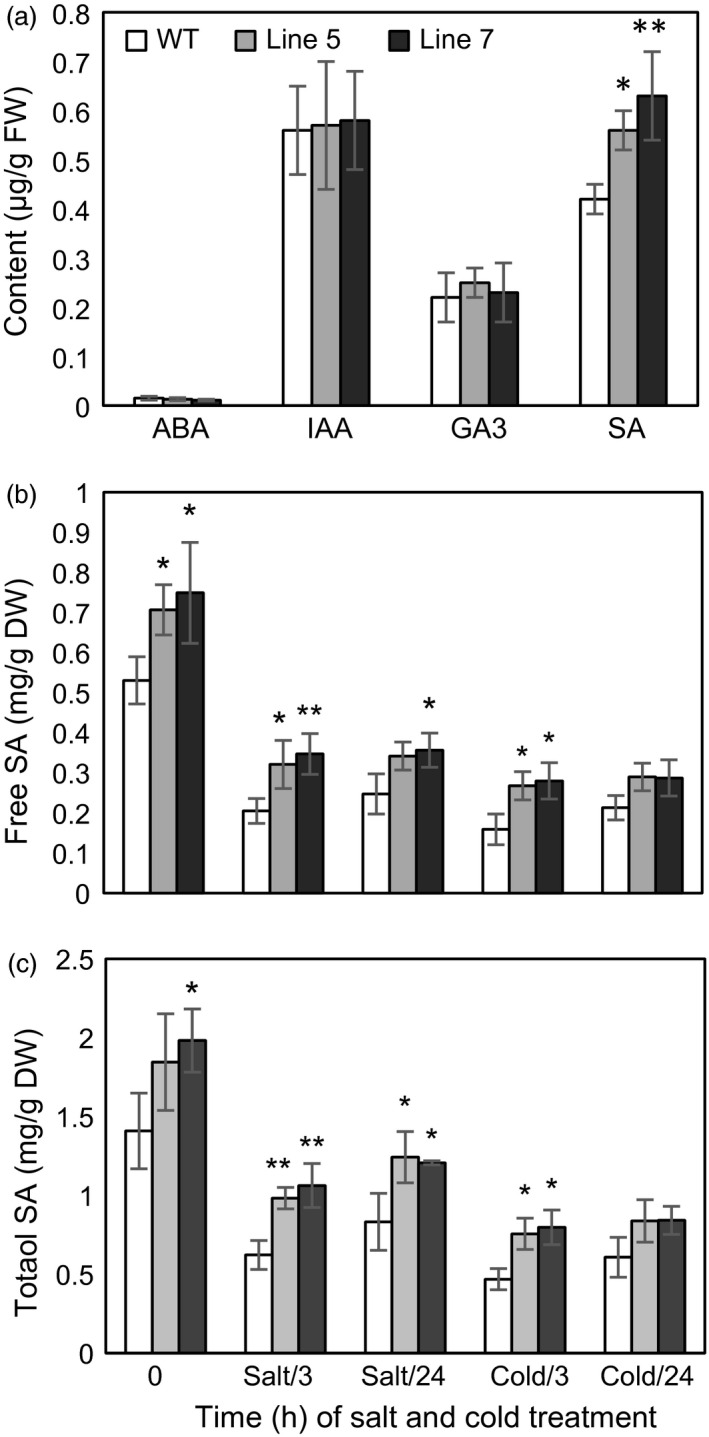
Hormone content in the leaves of the transgenic and wild‐type (WT) plants. (a) Contents of ABA, IAA, GA_3_ and SA in the leaves under normal growth conditions. (b) Total and free SA contents in the leaves under salt and cold treatments. Data are presented as means ± SD (*n* = 3). *Significant difference, *P *<* *0.05; **highly significant difference, *P *<* *0.01.

To examine whether *MdcyMDH* overexpression impacted hormone levels, the contents of ABA, IAA, GA_3_ and SA were determined. It was found that IAA and GA_3_ were not significantly altered in the transgenic plants. In contrast, the ABA level was slightly decreased, while the SA level was largely increased in the transgenic plants (Figure 8a). Free and total SA contents were further evaluated in the WT and transgenic plants under stress conditions. Compared to the WT controls, *MdcyMDH* overexpression elevated the free and total SA contents, and the increments reached a significant level at some time points under the stress treatments (Figure 8b,c). Free SA and total SA showed a fast decline induced by salt and cold temperature, but the decline was not intensified by the extension of the salt and cold treatments in the WT and transgenic plants. Moreover, *MdcyMDH* overexpression did not impact the ratio of free SA in the total SA (Figure 8b,c).

## Discussion

### cyMDH reaction favours malate synthesis under normal conditions and especially under abiotic stresses

The kinetic properties of recombinant cyMDH proteins showed that the cyMDH reaction favoured malate production *in vitro* in apple (Yao *et al*., 2011b), wheat (Ding and Ma, 2004) and pineapple (Cuevas and Podestá, 2000). Additionally, it was demonstrated that *MdcyMDH* promoted malate accumulation in *MdcyMDH*‐overexpressing apple callus (Yao *et al*., 2011b) and leaves (Figure 5a). Similarly, malate synthesis was promoted through the overexpression of *cyMDH* in tobacco and *Stylosanthes guianensis* (Chen *et al*., 2015; Wang *et al*., 2010). Therefore, regeneration of NAD^+^ and malate inside the cytosol was the most primary role of cyMDH, although the enzyme kinetics of the MDH reaction *in vivo* were affected by substrate/product ratios and the NAD^+^ redox state (Tomaz *et al*., 2010). Moreover, cyMDH reductive activity increased in response to cold and salt stresses in the WT and transgenic plants (Figure 4a), and cyMDH conferred superior manganese tolerance by increasing malate synthesis in *Stylosanthes guianensis* (Chen *et al*., 2015). Therefore, cyMDH primarily catalysed malate synthesis under stresses, and the reaction positively contributed to the tolerance of abiotic stresses.

Because cyMDH primarily catalysed the reductive reaction, more NADH was supposed to be converted into NAD^+^. However, a high ratio of NADH/NAD^+^ was found in the transgenic plants (Figure 7a), which can be explained, at least partly, by the following facts. First, NADH and NAD^+^ were used to generate other antioxidants and provide electron cofactors for other oxidoreductases and hence were tied to every other redox pair and large amounts of oxidoreductases (Hashida *et al*., 2009); second, large redox differences in the NAD(H) pools existed between cell compartments (Igamberdiev and Gardeström, 2003); third, *MdcyMDH* overexpression affected the expression of genes involved in NAD(H) metabolism, such as *quinolinate synthetase A* and *dihydrolipoamide dehydrogenase* (Figure 3c; Ceciliani *et al*., 2000). Therefore, the sole reaction catalysed by MdcyMDH could not determine the NAD(H) level of the cell, and an enhanced NADH/NAD^+^ might result from the wide metabolism modification mediated by *MdcyMDH* overexpression.

### *MdcyMDH* enhances the tolerance of the transgenic plants to cold and salt by improving the cell reducing power

Abiotic stresses, such as drought, salt and low temperature, can damage cells and lead to oxidative stress by producing certain deleterious chemical entities called ROS, which include H_2_O_2_, $O_{2}^{\cdot -}$, hydroxyl radical (OH^−^), etc. Enzymatic and nonenzymatic antioxidants can be employed to directly scavenge ROS and free radicals. In our previous study, *MdcyMDH* overexpression increased SOD and CAT activities in the transgenic apple callus and tomato plants (Yao *et al*., 2011b). In this study, the significantly increased nonenzymatic antioxidants (AsA and GSH) in the transgenic plants favoured the direct scavenging of ROS (Figure 7c,d; Noctor and Foyer, 1998). In contrast, AsA/DHA, GSH/GSSH, NADH/NAD^+^ and NADPH/NADP^+^ couples were placed at the heart of the cell's redox metabolism (Potters *et al*., 2010). Presently, the regulatory roles of cellular redox couples have predominantly come from studies using rather severe stress treatments that induced large amounts of ROS or programmed cell death (Potters *et al*., 2010). For example, severe salt stress caused a 10‐fold decrease in the GSH/GSSH ratio in *Arabidopsis* (Borsani *et al*., 2001). In contrast, the redox state and responses to mild stress remain a conundrum (Potters *et al*., 2010). Here, mild cold and salt stresses generated increased ratios of AsA/DHA, GSH/GSSG and NAD(P)H/NAD(P)^+^ (Figure 7a,e). Similarly, CO_2_‐induced heat stress alleviation can be associated with the reduction of oxidative stress through increasing AsA/DHA and GSH/GSSG ratios in tomato plants (Li *et al*., 2015). Therefore, it is suggested that the increased ratio of AsA/DHA, GSH/GSSG and NAD(P)H/NAD(P)^+^ contributed favourably to the mild oxidative stress tolerances.

Redox compounds are primarily derived from the mitochondrial and chloroplast metabolisms (Chew *et al*., 2003; Green and Fry, 2005). The modified chloroplast and mitochondrial metabolisms (Figures 5 and 6) might be one of the factors affecting the level of redox compounds in the transgenic plants. Similarly, it was reported that *mMDH* can alter photosynthetic, photorespiration and leaf respiration and affect ascorbate content in tomato and *Arabidopsis* (Nunes‐Nesi *et al*., 2005; Tomaz *et al*., 2010); plastid *NAD‐MDH* can alter glutathione levels and the respiration rate in *Arabidopsis* (Beeler *et al*., 2014). Additionally, modifications in the mMDH and chMDH activities were found in the *MdcyMDH* transgenic plants (Figure 4b,c). Therefore, it is speculated that MDHs from different cell compartments might cooperate to modify the chloroplast and mitochondrial metabolisms via malate valves. Moreover, another shuttle system (triose‐phosphate/3‐phosphoglycerate shuttle) between the chloroplast and the cytosol is suggested to be activated by *MdcyMDH* overexpression, based on the large expression modification of the key genes in this shuttle system, that is triose‐phosphate/phosphate translocator and glyceraldehyde‐3‐phosphate dehydrogenase (Figure 3c; Guo *et al*., 2012; Taniguchi and Miyake, 2012). Therefore, MdcyMDH might modify chloroplast and mitochondrial metabolisms and hence the redox state via different shuttle systems.

### *MdcyMDH* increases cold and salt tolerance by modifying the redox signal

The redox pairs within the cellular stress response network are more complex and do not simply act as a scavenger; redox metabolism and its associated signalling are important machinery during abiotic stress (Munné‐Bosch *et al*., 2013). It is suggested that ascorbate and glutathione pools have precise and distinct roles in redox signalling (Munné‐Bosch *et al*., 2013) and participate in the signal transduction pathways (Ramel *et al*., 2012). Changes in the glutathione content and in the ratio of GSH/GSSG clearly modify redox‐ and H_2_O_2_‐dependent gene regulation (Han *et al*., 2013). In this study, the increased ratios of AsA/DHA and GSH/GSSG (Figure 7e) may affect the redox signalling in the transgenic plants. On the other hand, ROS, such as H_2_O_2_ and $O_{2}^{\cdot -}$, are proposed to function in signalling between different organelles and the nucleus; the cytosol should be considered as a hub where cross‐communication between divergent ROS signals occurs (Choudhury *et al*., 2013). However, ROS accumulation at high levels causes oxidative stress leading to cell death (Choudhury *et al*., 2013). The dual function of ROS implies that its cellular concentration has to be tightly controlled (Apel and Hirt, 2004). In this study, the H_2_O_2_ level was increased, but $O_{2}^{\cdot -}$ was decreased by *MdcyMDH* overexpression (Figure 7f); this modification possibly altered the ROS signal, which was also suggested by the two largely up‐regulated *glyceraldehyde 3‐phosphate dehydrogenase* genes, related to H_2_O_2_ signal transduction (Figure 3c; Guo *et al*., 2012); the altered redox signalling might positively contribute to the increased cold and salt tolerance in the transgenic plants.

It is proposed that malate valves play an essential role in the regulation of catalase activity and the accumulation of a H_2_O_2_ signal by transmitting the redox state of the chloroplast to other cell compartments (Heyno *et al*., 2014). Therefore, the increase in H_2_O_2_ content in the transgenic plants may be influenced by the altered MdcyMDH‐mediated regulation of malate valves. Additionally, the expression of three peroxidases was largely down‐regulated (Figure 3c), suggesting a possible decrease in H_2_O_2_ scavenging, which led to the increase of H_2_O_2_ (Passardi *et al*., 2004). Moreover, $O_{2}^{\cdot -}$ can be rapidly converted to H_2_O_2_ by SOD1 or SOD2 (Murphy, 2009); the decreased $O_{2}^{\cdot -}$ (Figure 7f) might contribute to the increase of H_2_O_2_ in the transgenic plants.

### The promoted interaction of redox and SA favours the tolerance of the transgenic plants to cold and salt stresses

It is known that the SA signalling pathway is pivotal for enhanced salt and oxidative stress tolerance in *Arabidopsis* (Jayakannan *et al*., 2015). *MdcyMDH* overexpression might affect the stress tolerance of the transgenic plants via the SA signalling pathway based on the increased free and total SA (Figure 8b,c). SA is synthesized by two distinct pathways: The phenylalanine ammonia‐lyase pathway in the cytoplasm and the isochorismate (ICS1) pathway in the chloroplast. The ICS1 pathway is now thought to be the predominant pathway responsible for SA synthesis (Jayakannan *et al*., 2015). In the transgenic plants, an *ICS1* gene, marker gene in the ICS1 pathway and two *arogenate dehydratase* genes involved in the biosynthesis of phenylalanine were largely transcriptionally up‐regulated (Figure 3c), implying that *MdcyMDH* increased SA biosynthesis by promoting the above pathways. The defectiveness in the expression of the *ICS1* gene is hypersensitive to salt stress in *Arabidopsis* (Asensi‐Fabado and Munné‐Bosch, 2011), and salt‐ and cold‐induced *ICS1* expression was found in apple plants (Figure S2), implying that this pathway was essential for salt and cold tolerance in plants. Noticeably, two E3 ubiquitin‐protein ligase BAH1‐like genes were largely enhanced by *MdcyMDH* overexpression (Figure 3c), and they were largely induced by cold and salt (Figure S2); this type of gene is involved in the negative regulation of SA accumulation independent of *ICS1* (Kim *et al*., 2010) and might be one the factors decreasing the SA level when the transgenic and WT plants were subjected to stresses (Figure 8b,c). Taken together, *MdcyMDH* increased the SA level by several SA metabolism pathways including synthesis and degradation, which are likely to maintain the most suitable SA level under stress.

The modification of the redox state might be an important factor affecting the SA level. It has been found that an elevated NAD^+^ level did influence SA turnover and biosynthesis via downstream *ICS1* genes (Pétriacq *et al*., 2012). Other antioxidants, such as ascorbate and glutathione, are also central redox regulators of the SA signalling pathway (Bartoli *et al*., 2013). Moreover, ROS can influence the SA metabolism, and the H_2_O_2_‐dependent activation of SA‐dependent pathways is well characterized (Munné‐Bosch *et al*., 2013). On the other hand, the tight correlation between SA, H_2_O_2_ and GSH contents was observed in plants, implying an essential role of SA in the acclimation processes and in regulating the redox homeostasis of the cell (Mateo *et al*., 2006). Taken together, the complex network of cross‐communication between oxidants and antioxidants in the redox signalling hub and the different hormone signalling pathways regulates plant survival upon exposure to stress (Bartoli *et al*., 2013), which might be the key mechanism of *MdcyMDH*‐mediated abiotic stress tolerance.

Thus to conclude, *MdcyMDH* overexpression improved the tolerance of the transgenic apple plants to salt and cold conditions by the following possible mechanisms. First, the reducing power was enhanced, and the level of $O_{2}^{\cdot -}$ was decreased; second, changes in the redox couples and the level of H_2_O_2_ contributed favourably to stress tolerance as redox signals; third, the stress‐resistant pathway mediated by SA was strengthened; finally, the widely modified gene expression in the transgenic plants implicated that redox compounds and SA participated in the chloroplast‐to‐nucleus and mitochondria‐to‐nucleus retrograde communication, which served to coordinate gene expression in the nuclear and cytoplasmic genomes and played an important role in the stress acclimation of plants (Cela *et al*., 2011; Ramel *et al*., 2012).

## Experimental procedures

### Plant materials and growth conditions

‘Gala’ apple *in vitro* shoot cultures were used for the gene expression assays, genetic transformation and other analyses. The ‘Gala’ cultures were grown on MS subculture media containing 0.6 mg/L of 6‐BA and 0.2 mg/L of IAA at 25 °C under a 16‐h photoperiod with a light intensity of 600 μmol/m^2^/s. Three‐year‐old pot‐cultured WT and transgenic apple trees were used for the salt treatment, photosynthesis and chlorophyll fluorescence assays.

### RNA extraction and gene expression analysis with real‐time quantitative PCR

Total RNAs were extracted from apple *in vitro* shoot cultures using TRIzol Reagent (Invitrogen, Carlsbad, CA), according to the manufacturer's instructions. Two micrograms of total RNAs was used to synthesize first‐strand cDNA. For real‐time quantitative PCR, the specific primer pairs are given in Table S1. The reactions were performed using SYBR Green MasterMix (SYBR Premix EX Taq TM, Dalian, China), as described by the manufacturer. Real‐time quantitative PCRs were performed using a BIO‐RAD iQ5 (Hercules, CA) instrument. The expression values obtained were normalized against *18s* rRNA by the cycle threshold (*C*_t_) $2^{-\Delta \Delta C_{t}}$ method (Software IQ5 2.0) (Bio‐Rad, Hercules, CA).

### *Agrobacterium*‐mediated genetic transformation of *MdcyMDH* into apple *in vitro* shoot cultures

The *MdcyMDH* ORF was obtained by PCR using the forward primer 5′‐GGTCTAGAATGGCGAAAGAACCAGTTC‐3′ and the reverse primer 5′‐CGTCGACAGTCGAAGTGTCCGAATAGAAT‐3′. The forward primer contained a *Xba* I digestion site, and the reverse primer contained a *Sal*I site (both underlined). Subsequently, the PCR product was cloned into the pMD18‐T vector (TaKaRa, Dalian, China). *MdcyMDH* was double‐digested with *Xba*I and *Sal*I, and then ligated into the pBI121 vector, downstream of the *CaMV 35S* promoter. The resultant constructs were introduced into the *Agrobacterium* strain LBA4404 and transformed into apple leaves from *in vitro* shoot cultures, as described by our previous study (Feng *et al*., 2012). The specific primers from *35s* (5′‐GACGCACAATCCCACTATCC‐3′) and *MdcyMDH* (5′‐GGATCCAGAGAGGCAAGAGTA‐3′) were used in PCR to determine whether *MdcyMDH* was integrated into the apple plants. A plasmid DNA containing the *pBI121*::*MdcyMDH* construct and H_2_O was used as positive and negative controls.

### Enzyme extraction and isolation of mitochondria and chloroplast

Cytosolic enzyme extractions were performed according to our previous study (Yao *et al*., 2011b). Mitochondria and chloroplast were isolated mechanically from 15 and 10 g of leaves of apple *in vitro* shoot cultures, respectively, and then purified by Percoll gradient centrifugation as described by Osmond and Makino (1991). Mitochondrial integrity was detected by measuring the cytochrome *c* oxidase activity with and without 0.05% Triton X‐100. In the tested samples, the membrane integrity exceeded 86%. The intactness of the purified chloroplast fraction was over 84% as judged by the ferricyanide test. The purities of the cytosolic, mitochondria and chloroplast fractions were verified by measuring the activities of maker enzymes located in other organelles (Table S2). Protein amounts were quantified using dye‐binding assay with bovine serum albumin (Bradford, 1976).

### MDH activity assays and malate‐dependent respiratory rates

MDH reductive activity was measured in 1 mL of a reaction mixture containing 50 mm Tris–HCl (pH 7.8), 2 mm MgCl_2_, 0.5 mm EDTA, 0.2 mm NADH, 2 mm OAA and 50 μL of extract at 30 °C. The reaction was initiated by adding OAA. MDH oxidative activity was measured in 1 mL of a reaction mixture containing 50 mm Tris–HCl (pH 8.9), 2 mm MgCl_2_, 0.5 mm EDTA, 0.2 mm NAD^+^ and 25 mm malate. The reaction was initiated by adding malate. For each reaction, 5 min of spectrophotometric change at 340 nm was monitored automatically at 40‐s intervals, and the activities were calculated according to the slopes of the obtained lines. The assay conditions were not optimized with respect to the concentration of each component of the reaction mixture. However, linear relationships between the activity and time and amount of the extract were found.

Malate‐dependent respiratory rates on isolated intact mitochondria were performed according to the method of Tomaz *et al*. (2010).

### Determination of malate content

Malate content was determined using a capillary electrophoresis system (Beckman P/ACE, Palo Alto, CA) as described in our previous study (Yao *et al*., 2007).

### Measurement of photosynthesis and chlorophyll content

Photosynthesis was measured using a portable photosynthesis system (CIRAS‐2; PPS Co. Ltd., Hitchin, UK). Photosynthesis was measured three times for each selected leaf, so that nine measurements were made for each plant. Measurements were made between 8:30 and 11:00 h on a sunny day. During each measurement, CO_2_ concentration was maintained at 396 ± 21 μL/L by the CIRAS system at an air temperature of approximately 25 °C and a relative humidity of 85 ± 0.9%. Chlorophyll content was determined according to the methods described by Zhu *et al*. (1990).

### Measurement of chlorophyll fluorescence

Chlorophyll fluorescence was measured with a FMS‐2 pulse‐modulated fluorometer (Hansatech, Norfolk, UK). The light‐fluorescence measurement protocol was as follows: The light‐adapted leaves were continuously illuminated by actinic light at 100 μmol/m^2^/s from the FMS‐2 light source; steady‐state fluorescence (*F*_s_) was recorded after 2 min of illumination; and 0.8 s of saturating light of 8000 μmol/m^2^/s was imposed to obtain a maximum fluorescence in the light‐adapted state (*F*_m_). The actinic light was then turned off, and the minimum fluorescence in the light‐adapted state (*F*_0_) was determined by 3 s of illumination with far‐red light. The following parameters were then calculated as follows (Maxwell and Johnson, 2000):

$$\text{Quantum yield of PSII},\Phi \text{PSII}=\left(F_{m}-F_{s}\right)/F_{m}$$

$$\begin{array}{cc} \text{Electron transport rate}, & \text{ETR}=\Phi \text{PSII}\times \text{PFD}\times 0.5\times 0.84,\\ & \text{PFD}=1000 \end{array}$$

$$\text{Maximum quantum yield of PSII},\;F_{v}/F_{m}=1-\left(F_{0}/F_{m}\right).$$

### Extractions and assays of redox couples and ROS

Extractions and determinations of the four redox couples, that is NADH/NAD^+^, NADPH/NADP^+^, reduced ascorbate (AsA)/oxidized ascorbate (DHA) and reduced glutathione (GSH)/oxidized glutathione (GSSG), were performed as described by Queval and Noctor (2007). Hydrogen peroxide (H_2_O_2_) and superoxide radical ($O_{2}^{\cdot -}$) were extracted and measured according to the methods described by Zhu *et al*. (1990).

### Hormone extraction and determination

Lyophilized leave (0.5 g) was ground into a powder. The powdered samples were extracted three times in 5 mL of cold 80% methanol (v/v) mixed with 30 μg/mL of sodium diethyldithiocarbamate. The extracts were centrifuged at 8000 ***g*** and 4 °C for 10 min. The supernatant was concentrated to dryness under vacuum. The residue was dissolved in 4 mL of 0.4 m phosphate buffer (pH 8.0). The solution was mixed with 4 mL of trichloromethane to remove the pigment. After centrifuging at 8000 ***g*** and 4 °C for 10 min, the aqueous phase was collected and supplemented with polyvinylpyrrolidone to remove the phenolics, and then centrifuged at 8000 ***g*** and 4 °C for 10 min. The supernatant was extracted with 4 mL of ethyl acetate (pH 3.0) twice. The upper phase was collected and concentrated to dryness under vacuum. The residue was dissolved in 1 mL of 0.5% (v/v) acetic acid/methanol (55 : 45, v/v) and finally filtered through a 0.45 μm filter. The obtained solution was used to determine ABA, GA3, IAA and SA. Extractions of free and conjugated SA were performed as described by Fragnière *et al*. (2011).

The extracted hormones were quantified using a LC‐ESI‐MS (Thermo, San Jose, CA) instrument. Ten microlitres of the sample was injected into a Thermo Scientific Hypersil Gold column (50 × 2.1 mm, 1.9 μm) in the Thermo Scientific Ultimate 3000 HPLC system (Thermo, San Jose, CA). The HPLC solvents were as follows: A, 0.04% acetate acid in water and B, methanol (0.4 mL/min). The two mobile phases were used in the gradient mode under the following time/concentration (min/%) of B: 0.0/20, 0.5/20, 2.5/90, 3.5/90, 3.6/20 and 5.0/20. Detection and quantification were performed using the TSQ Quantum Access MAX system (Thermo). SA was detected in the ESI negative mode and selected reaction monitoring with the following parameters: parent mass by charge (*m*/*z*) of 263.1, daughter mass by charge (*m*/*z*) of 153.0 and collision energy of 14 eV. The parameters for the ion source were set as follows: ion spray voltage of 3000 V, temperature of 350 °C, collision gas pressure of 1.5 mTorr, sheath gas of 25 arbitrary units and auxiliary gas of 15 arbitrary units. The amount of free and conjugated SA was calculated in mg/g DW with reference to the amount of internal standard (ortho‐anisic acid).

### Sequence‐based DGE

Sample preparation and sequencing were performed with the Illumina Gene Expression Sample Prep kit and Solexa Sequencing Chip (flowcell) according to the manufacturer's introductions on the Illumina Cluster Station and Illumina HiSeq™ 2000 system (Illumina Inc., San Diego, CA). The sequencing lengths of raw reads were 49 bp in each line of the flowcell tunnel. The raw sequences were transformed into 17 bp clean tags, and tag counting was carried out using the Illumina Pipeline.

All tags were annotated using the apple genome database (http://genomics.research.iasma.it/). Briefly, a preprocessed database containing all possible CATG + 17‐base tag sequences was created. All clean tags were mapped to the reference sequence, and only 1 bp mismatch was considered. Clean tags mapped to the reference sequences from multiple genes were filtered. The remainder clean tags were designed as unambiguous clean tags. The number of unambiguous clean tags for each gene was calculated, and then normalized to TPM (number of transcripts per million clean tags).

Finally, a rigorous algorithm developed by the Beijing Genomics Institute (BGI) referring to ‘the significance of digital gene expression profiles’ (false discovery rate < 0.001) (Audic and Claverie, 1997) was used to identify differentially expressed genes between two samples, and absolute value of log_2_ratio ≥1 (minimum of twofold difference) was used as the threshold to judge the significance of gene expression differences.

## Conflict of interest

The authors declare no conflicts of interest.

## Supporting information


**Figure S1** Conserved domains (a) and sequence alignment (b) of MdcyMDH and the other four cyMDH isoforms.
**Figure S2** Expression modifications of the selected DGE genes related to SA biosynthesis and signalling, mitochondrial and chloroplast metabolism, redox and abiotic stress tolerance in response to cold and salt treatments.
**Table S1** Primer sequence for real‐time quantitative RT‐PCR.
**Table S2** Marker enzyme activities in isolated cytosolic and mitochondrial fractions of the leaves under normal growth conditions.
