# Supplementary material for: The enhancement of tolerance to salt and cold stresses by modifying the redox state and salicylic acid content via the cytosolic malate dehydrogenase gene in transgenic apple plants
Source: Plant Biotechnol J. 2016 Mar 29;14(10):1986–97. doi: 10.1111/pbi.12556 (PMC5043475; doi:10.1111/pbi.12556)
Supplement: Supplementary file 1 — Figure S1 Conserved domains (a) and sequence alignment (b) of MdcyMDH and the other four cyMDH isoforms. Figure S2 Expression modifications of the selected DGE genes related to SA biosynthesis and signalling, mitochondrial and chloroplast metabolism, redox and abiotic stress tolerance in response to cold and salt treatments. Table S1 Primer sequence for real‐time quantitative RT‐PCR. Table S2 Marker enzyme activities in isolated cytosolic and mitochondrial fractions of the leaves under normal growth conditions. [file PBI-14-1986-s001.docx]

**Supplementary data**

**(a)**


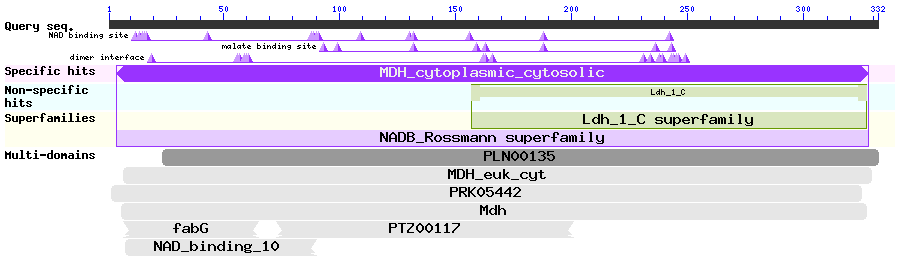


MdcyMDH


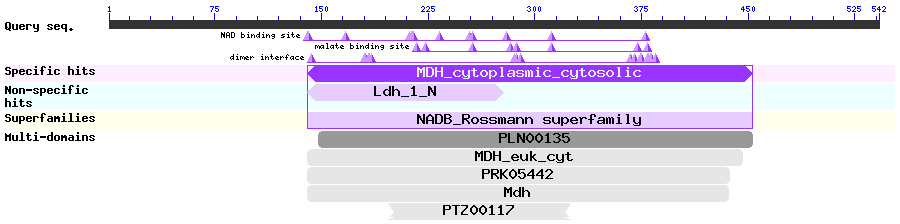


MDP0000170418


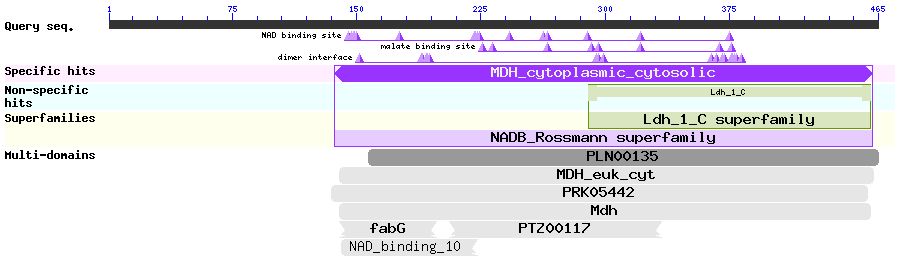


MDP0000174740


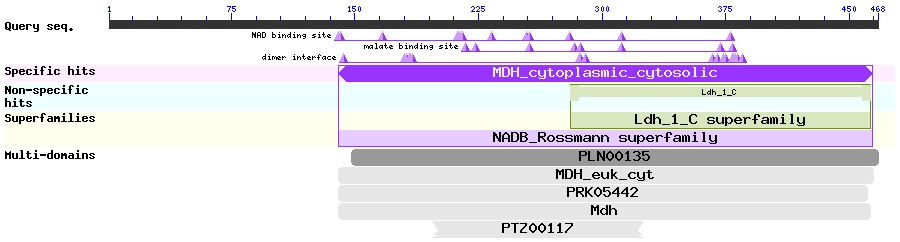


MDP0000197620


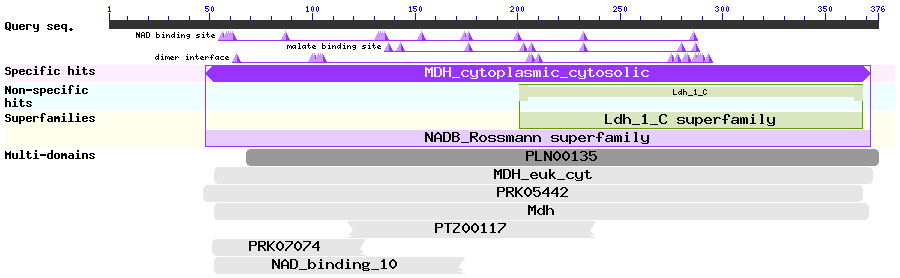


MDP0000926135

**(b)**

**Figure S1**. Conserved domains (a) and sequence alignment (b) of MdcyMDH and the other four cyMDH isoforms.

Conserved domains were obtained by online analysis (<http://www.ncbi.nlm.nih.gov/Structure/cdd/wrpsb.cgi>), and sequence alignment was performed using DNAman software.

Salt/12 h

Salt/3 h

Cold/12h h

Cold/3 h

WT L5 L7 WT L5 L7 WT L5 L7 WT L5 L7

**
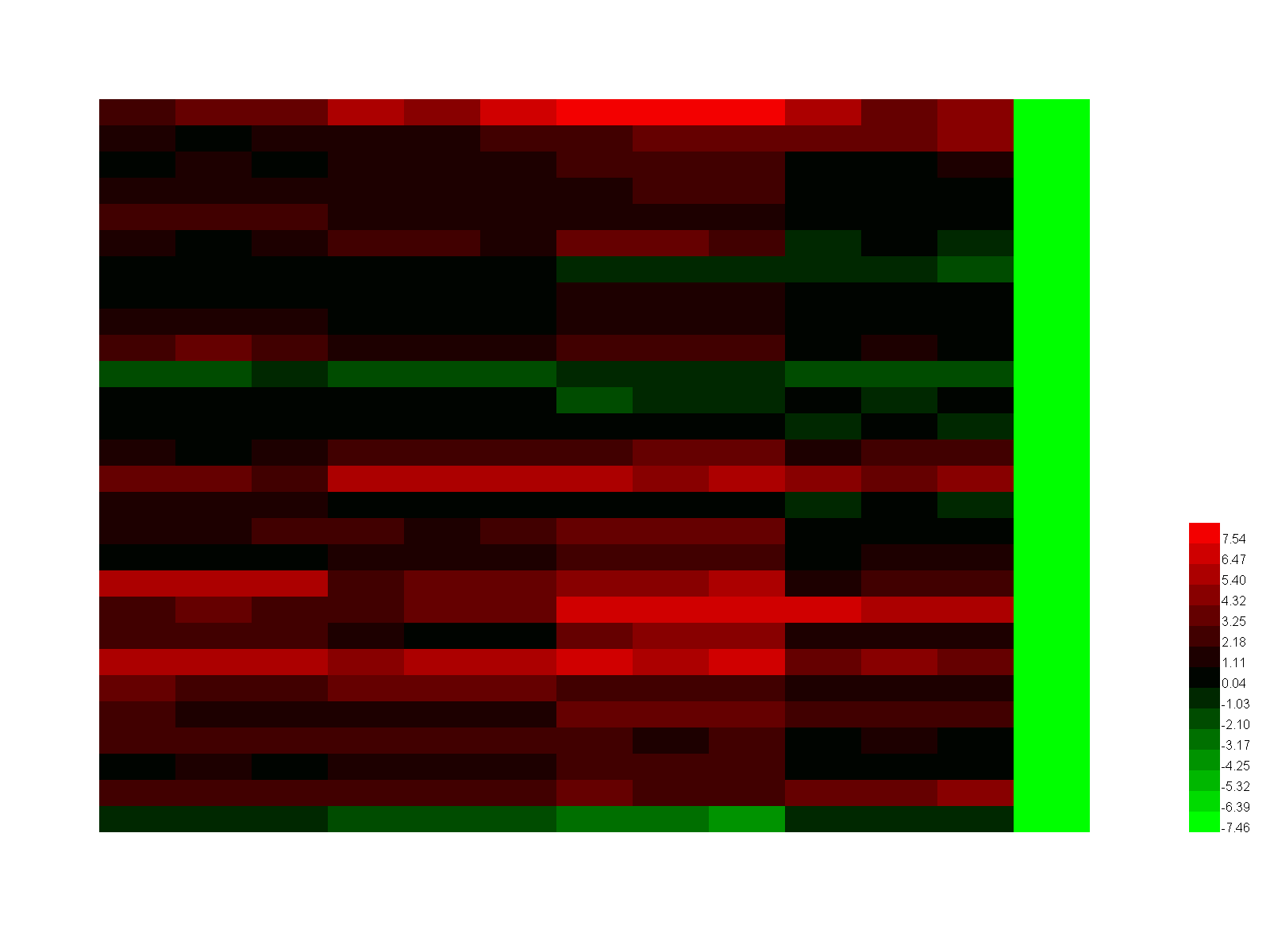
**

Abiotic stress tolerance

Redox

Chloroplast

metabolism

Mitochondrial

metabolism

SA biosynthesis and signal transduction

E3 ubiquitin-protein ligase BAH1-like

E3 ubiquitin-protein ligase BAH1-like

Isochorismate synthase

Arogenate dehydratase

Arogenate/prephenate dehydratase

Chitinase-like protein 2

3-hydroxy-3-methylglutaryl coenzyme A reductase

Mitochondrial aldehyde dehydrogenase 2

Organic acid transmembrane transporter activity

Ribulose bisphosphate carboxylase/oxygenase activase

Chloroplast small heat shock protein 1

Photosystem II CP47 chlorophyll apoprotein

Photosystem I P700 apoprotein A2

Ent-kaurene synthase A

Quinolinate synthetase A-related family protein

Triose phosphate/phosphate translocator, chloroplastic

Glyceraldehyde-3-phosphate dehydrogenase

Glyceraldehyde-3-phosphate dehydrogenase

Monodehydroascorbate reductase, cytoplasmic isoform 2

Peroxidase P7

Cationic peroxidase 2

Peroxidase 16

AP2/ERF domain-containing transcription factor

ERF transcription factor 4

CBF/DREB transcription factor

HVA22

HVA22

delta-1-pyrroline-5-carboxylate dehydrogenase 1

MDP0000150030

MDP0000190623

MDP0000283280

MDP0000360526

MDP0000672088

MDP0000424978

MDP0000312032

MDP0000859857

MDP0000144687

MDP0000321244

MDP0000214382

MDP0000551952

MDP0000441013

MDP0000305417

MDP0000274925

MDP0000144687

MDP0000285734

MDP0000527995

MDP0000152184

MDP0000770103

MDP0000206714

MDP0000142485

MDP0000932292

MDP0000689946

MDP0000451365

MDP0000294329

MDP0000594632

MDP0000465381

**
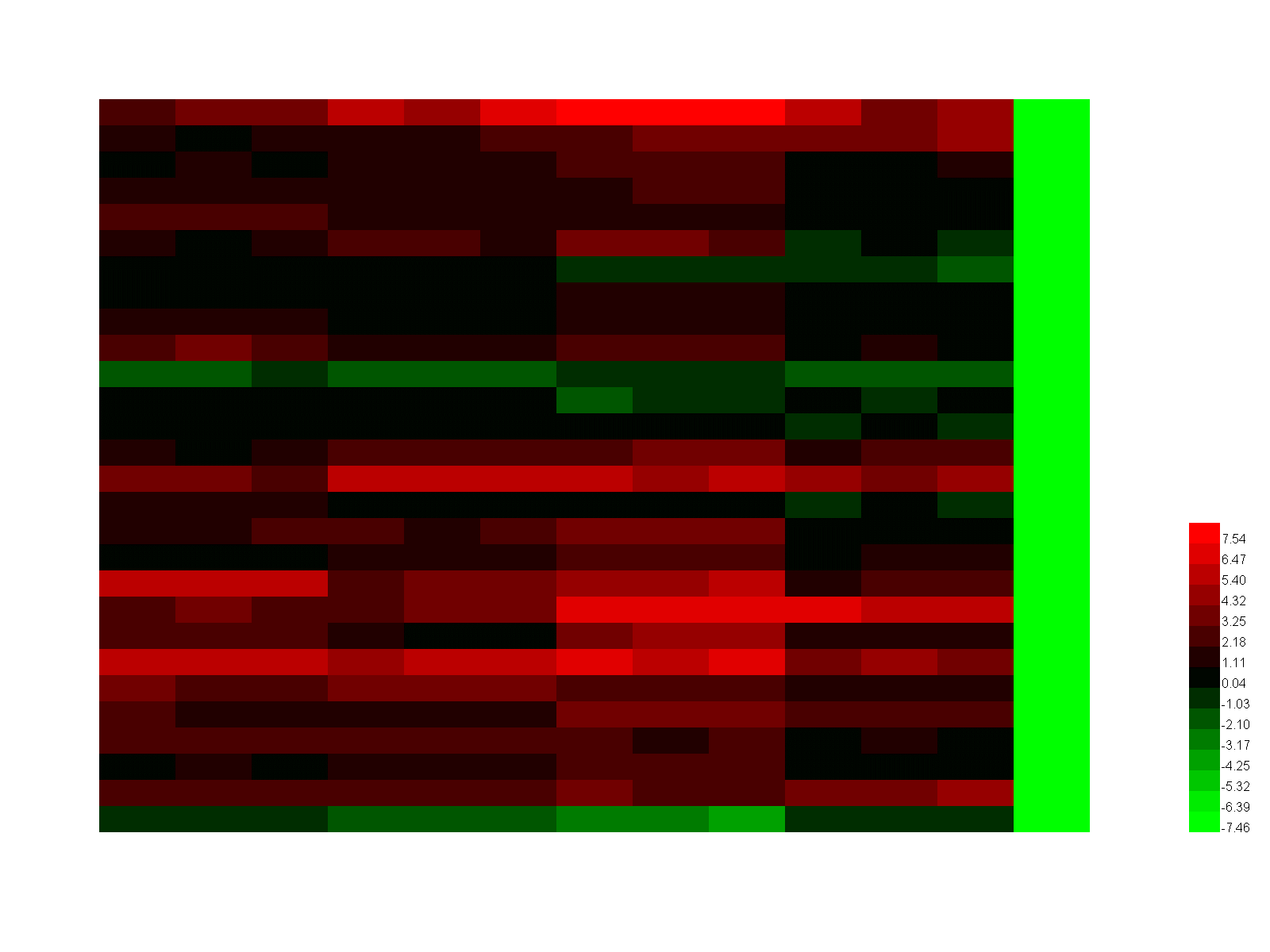
**

**Figure S2** Expression modifications of the selected DGE genes related to SA biosynthesis and signaling, mitochondrial and chloroplast metabolism, redox, and abiotic stress tolerance in response to cold and salt treatments.

Fold-change from qRT-PCR is calculated by comparing the relative expression values of the selected genes in the stress-treated and 0-h control plants. Data are presented as the means of three replicates.

**Table S1** Primer sequence for real-time quantitative RT-PCR

| **Gene target** | **Forward primer (5'-3')** | **Reverse primer (5'-3')** |
| --- | --- | --- |
| *MdcyMDH* (DQ221207) | TCTCGAGGAAGAAGTTGGATGC | TCAGCTGCTTTTCTCACACATTG |
| MDP0000150030 | CAAAGCAGCACTGGGAAT | TAGCAAGGTTCAAGGAGCC |
| MDP0000190623 | GGCTATCTCACCCTGGAAG | TGAAGCTCCTCGTGCATAAGT |
| MDP0000283280 | CTCTCTACAGAATGTCCGCTT | ATCTCCCAAAGTCCACGA |
| MDP0000360526 | GTGTCGTCGACGGCTTC | GTCATCTCGCATCCTCAAGC |
| MDP0000672088 | GAGTCACTTCGAGTACCTGTTCT | CAGCACCCTCAAGAACGA |
| MDP0000424978 | TCAGTTTGTGGCAAGGGT | AAGATGGCTTTGATAGGGC |
| MDP0000274925 | TCTGTGAAGAGGGACAACAAG | GTAGGCAATCCAAAGTTCCA |
| MDP0000144687 | CACTAACGCATGCAGTCGG | TGGCAACACCAGCGATT |
| MDP0000285734 | AAGCCCAAGCGTTACCAA | GGATGTTGCGTTGATAATGC |
| MDP0000527995 | GTCTCAGTCGACTTTAGGTGC | GACAATGTCAGCCAAATCAAC |
| MDP0000142485 | GATGTGTCCAACTCGAGTGG | CCTTGCTGAAGATTCTGGAAG |
| MDP0000689946 | GAACCCACCAAGAACGTC | GCTGCTCGATCATATGCC |
| MDP0000451365 | TATGATGACGACGACTGGG | CCAAAGAAACCCTAATGCG |
| MDP0000294329 | GGCTGATAGAGAAAGGAGCA | GATGTATGTGTTGGCTTAGCAG |
| MDP0000465381 | GAACTCTGCCTGCATTATCC | CGACGCTGATGATGCCT |
| MDP0000312032 | CTGAACCTGCTCGGAGTG | ACAAGCTGTCCAGCTGAAATAG |
| MDP0000859857 | GGACGTTGATAAGGTTGCG | TGCCATCTCAACAGCCTTATC |
| MDP0000321244 | TACCATTGGATCTGTCAACAG | TTGGAACTGAAGCACTGCT |
| MDP0000214382 | CAGCCAAGAGCTATGGGAG | GCCTTTGGAATGGTAATGTAC |
| MDP0000551952 | GGTCCTGGAATATGGGTGTC | TCCCTGCTGCAATATGATGA |
| MDP0000441013 | ATGGTCCAGCGTTCAATG | CTCCAGGTCCTATTGTTAAGAAT |
| MDP0000305417 | GACGACATAGAAGGAGATGAAG | GTCGTATGCCGAGATTGATATC |
| MDP0000152184 | CGCATTGACAGTTAGCCAG | ATACGCGAACGCTGCTA |
| MDP0000144687 | ACCACTAACGCATGCAGT | CGATGGCAGTTCCAATC |
| MDP0000770103 | CGGACCAGGAACTCTTCAA | CTCATCTTCACCATGGCTG |
| MDP0000932292 | CAGCTGGAACTGGAAGCT | CGTTGCCAAATCCATGTC |
| MDP0000594632 | TTCAGCAAGCCGGATGAC | CTCGATCAACCTTGCTTATG |
| *18s* rRNA (CV826359) | AAACGGCTACCACATCCA | CACCAGACTTGCCCTCCA |

|  |  | Enzyme activity in total  soluble protein fraction | | | |  | Enzyme activity in cytosolic  protein fraction | | | Enzyme activity in  mitochondrial fraction | | | Enzyme activity in  chloroplast fraction | | |
| --- | --- | --- | --- | --- | --- | --- | --- | --- | --- | --- | --- | --- | --- | --- | --- |
| Localization | Marker enzyme | WT | Line 5 | Line 7 | |  | WT | Line 5 | Line 7 | WT | Line 5 | Line 7 | WT | Line 5 | Line 7 |
| Cytosolic | Alcohol dehydrogenase | 643.17± 90.42 | 656.67± 59.97 | 639.25± 77.16 |  | **1390.08± 106.77** | | **1339.62 ±152.67** | **1569.25± 171.08** | 8.73± 1.05 | 8.05± 0.67 | 9.91± 1.33 | 12.52 ±2.56 | 11.50 ±1.78 | 14.75 ±1.07 |
| Mitochondria | Cytochrome  c oxidase | 5.85±0.38 | 5.94± 0.64 | 6.49±0.48 | |  | 0.25± 0.04 | 0.27±0.04 | 0.31±0.03 | **417.25±39.64** | **451.69±40.19** | **432.87±50.19** | 0.11 ±0.02 | 0.23 ±0.02 | 0.09 ±0.01 |
| Peroxisome | Hydroxypyruvate reductase | 346.13± 60.44 | 339.51± 40.18 | 349.21 ±53.46 | |  | 11.95± 1.33 | 13.56± 1.16 | 15.02± 0.97 | 5.98± 0.73 | 6.09± 0.85 | 6.64± 0.44 | 12.05 ±0.97 | 17.25 ±1.56 | 14.28 ±5.04 |
| Plastid | Alkaline pyrophosphatase | 124.41  ± 13.29 | 132.51  ± 16.08 | 109.45 ±12.58 | |  | 4.87± 0.61 | 3.32  ± 0.45 | 4.23  ±0.62 | 7.51  ±0.66 | 6.79± 0.81 | 6.92  ±0.91 | **418.44 ±15.26** | **465.58 ±35.17** | **444.48 ±19.51** |

**Table S2** Marker enzyme activities in isolated cytosolic and mitochondrial fractions of the leaves under normal growth conditions

Note: Alcohol dehydrogenase was used as a cytosolic marker enzyme, and cytochrome c oxidase was as a mitochondrial membrane marker (Cvetić *et al*., 2008). Hydroxypyruvate reductase was used as a marker for peroxisome (Cvetić *et al*., 2008) and alkaline pyrophosphatase was used as a marker for plastid (Farre *et al*., 2001). Marker enzyme activities are expressed in nmmol.mg^-1^ protein min^-1^ for alcohol dehydrogenase and hydroxypyruvate reductase, in unit mg^-1^ protein for cytochrome c oxidase and in nmol PPi min^-1^ g^-1^ fresh weight for alkaline pyrophosphatase.

From Table S2, it was found that the cytosolic protein fraction exhibited a high cytosolic marker enzyme activity but low marker enzyme activities of the other organelles, i.e., less than 4.8, 4.3 and 3.9% of the total soluble activities of cytochrome c oxidase, hydroxypyruvate reductase and alkaline pyrophosphatase, respectively. Thereby, cytosolic extracts primarily consisted of cytosolic proteins. Similar results were found for the mitochondrial and chloroplast extracts.

Cvetić, T., Veljović-Jovanović, S. and Vučinić, Ž. (2008) Characterization of NAD-dependent malate dehydrogenases from spinach leaves. *Protoplasma*, **232**, 247-253.

Farre, E.M., Bachmann, A., Willmitzer, L. and Trethewey, R.N. (2001) Acceleration of potato tuber sprouting by the expression of a bacterial pyrophosphatase. *Nat. Biotechnol.* **19**, 268–272.
